# Hydration Strategies in Ultra-Endurance Running: A Narrative Review of Programmed Versus Thirst-Driven Approaches

**DOI:** 10.3390/nu17223526

**Published:** 2025-11-11

**Authors:** Shawn C. Wierick, Rosie I. Perez, Xiujing Zhao, Brendon P. McDermott

**Affiliations:** Heat & Hydration Optimization (H_2_O) Laboratory, Exercise Science Research Center, University of Arkansas, Fayetteville, AR 72701, USA; scwieri@uark.edu (S.C.W.); rosiep@uark.edu (R.I.P.); xiujingz@uark.edu (X.Z.)

**Keywords:** ultramarathon, programmed fluid intake, thirst-driven fluid intake, dehydration, exercise-associated hyponatremia, endurance performance, sports nutrition

## Abstract

Background/Objectives: Ultra-endurance running (UER) presents unique hydration challenges due to prolonged duration, variable terrain, environmental extremes, and gastrointestinal limitations. Athletes often use either programmed fluid intake (PFI), which prescribes fluid volumes based on estimated sweat rate, or thirst-driven fluid intake (TDFI), which relies on internal cues. This review examines the effectiveness and limitations of each strategy in the context of UER performance and safety. Methods: A narrative review was conducted using a targeted selection of peer-reviewed studies. Both laboratory- and field-based research were included to evaluate the physiological rationale, practical feasibility, and outcomes associated with PFI and TDFI. A total of six studies (five field-based ultra-endurance and one laboratory-based endurance protocols) were included for narrative synthesis. Results: Laboratory trials support PFI for preserving plasma volume, reducing cardiovascular strain, and improving performance in prolonged exercise under controlled conditions. However, real-world ultra-endurance events often involve environmental and logistical challenges that limit the applicability of rigid hydration strategies. Field studies demonstrate that TDFI is safe and effective for many experienced athletes, with no increased incidence of exercise-associated hyponatremia or measurable performance impairment, even with moderate body mass loss. Still, TDFI may underperform in individuals with high sweat rates or impaired thirst perception. Conclusions: Neither strategy seems universally superior. A hybrid model that integrates individual sweat testing, environmental context, and responsiveness to internal cues may offer the most practical and effective hydration approach in ultra-endurance running. Continued research is needed to validate hydration strategies under field conditions and to inform personalized, performance-oriented guidelines.

## 1. Introduction

Ultra-endurance running (UER) represents one of the most physiologically demanding categories of endurance sports. These events are commonly defined as continuous efforts that exceed the standard marathon distance (42.195 km) or last six hours or more. UER events include trail ultramarathons, mountain races, and multi-day stage events, often held under extreme environmental conditions [1,2]. The prolonged nature of these events challenges nearly every aspect of human physiology, with hydration status playing a critical role in thermoregulation [3,4], cardiovascular stability [5], gastrointestinal function [6], and overall performance [7,8].

Maintaining hydration during UER is inherently complex, and dehydration is common, regardless of other factors. Athletes are exposed to highly variable terrain, temperatures, humidity, and elevation, all of which influence sweat rate, fluid needs, and often limit hydration resources. At the same time, logistical constraints such as aid station spacing, gut tolerance, and fatigue can limit intake despite apparent need. Traditionally, hydration recommendations have emphasized programmed fluid intake (PFI), which encourages athletes to replace fluids based on measured sweat rates and avoid exercise-induced body mass losses exceeding 2% [3,4]. However, this approach has been increasingly questioned in recent years due to concerns about overhydration, exercise-associated hyponatremia (EAH), and a lack of feasibility in real-world UER settings [9,10].

In contrast, thirst-driven fluid intake (TDFI) seems to have gained favor as an easier, self-regulated strategy. Advocates argue that it better reflects individual needs and minimizes risks of overconsumption and sodium dilution, particularly in long-duration events with variable pacing and environmental variations [6,10]. While field studies support TDFI’s effectiveness and safety, critics note its limitations in extreme environments or among less-experienced athletes, where thirst cues may be delayed or suppressed. Therefore, thirst alone may be insufficient in many UER environments to guide adequate fluid replacement.

The resulting debate between PFI and TDFI reflects broader questions about the applicability of laboratory-driven hydration strategies in dynamic, self-paced competition. To date, few studies have directly compared these approaches in ultra-endurance settings, and existing guidelines often fail to account for the vast inter-individual variability and logistical challenges that define this sport. Furthermore, previous reviews have critically examined these strategies, but not specifically on the UER population.

This review critically examines the current literature addressing the effectiveness of programmed hydration versus drinking to thirst in UER. Using a participant, intervention, comparison, and outcome framework, we explore how hydration strategy influences performance, hydration status, and athlete safety during prolonged competition. Relevant studies were identified through searches of PubMed, Scopus, and Google Scholar using combinations of the terms ‘ultra-endurance’, ‘hydration’, ‘dehydration’, ‘programmed’, ‘thirst-driven’, ‘ad libitum’, and ‘performance’. Only full-text, peer-reviewed human studies published through June 2025 were included. Reference lists of identified papers were also reviewed to capture additional relevant sources. Both laboratory- and field-based studies were evaluated to provide a comprehensive overview of current evidence. By synthesizing findings from these studies, this review aims to clarify best practices and propose a path forward that balances evidence-based guidance with athlete autonomy and context-specific flexibility.

## 2. Hydration Challenges in Ultra-Endurance Running

UER presents unique physiological, environmental, and logistical challenges for hydration and fluid balance. Events lasting greater than 6 h, ranging from desert and mountain settings to 24 h competitions and multi-day stage races, place sustained stress on thermoregulation, fluid balance, and gastrointestinal tolerance [2]. Maintaining euhydration throughout these events requires more than simply replacing sweat losses; it demands adaptive strategies that account for variable terrain, prolonged exposure to environmental extremes, and fluctuating exercise intensity. While hydration primarily comes from fluid intake, foods with high water and electrolyte content, such as soups, fruits, and gels, can also contribute to overall fluid balance, particularly during prolonged events, when fluid access or tolerance is limited [11,12]. These events also involve long climbs, technical descents, and aid station transitions, imposing varied physiological demands [2]. For example, runners in mountainous ultramarathons may ascend and descend thousands of meters, encountering dramatic shifts in temperature and humidity between high-elevation cold zones and sun-exposed valleys. Belinchon-deMiguel et al. [13] documented such variability in a tropical mountain ultramarathon, where runners experienced both dehydration and sodium imbalance despite high fluid intake. In contrast, desert ultramarathons often involve prolonged exposure to dry heat and limited shade, while tropical races present sustained heat and humidity that strain thermoregulation and fluid turnover [1,14,15].

One of the most difficult challenges presented is the wide inter-individual variability in sweat rate and composition, which may range from 0.4 to over 2.5 L per hour and varies based on acclimatization, ambient temperature, and exercise intensity [3]. Sodium concentration of sweat also varies widely between athletes and across environments, compounding the difficulty in estimating sodium and other electrolyte losses [16]. Furthermore, field studies have demonstrated that even in cool environments with frequent fluid access, athletes commonly replace less than half of their sweat losses, either due to low thirst, limited gut tolerance, or misperception of fluid needs [17].

Another complicating factor is the changing composition of body mass during UER events, which makes interpreting fluid loss from body weight reductions alone misleading. As Hoffman [10] and Noakes [9] both argue, body mass losses reflect not only sweat loss but also fuel oxidation, glycogen-associated water release, and respiratory loss, none of which reflect true dehydration. Consequently, efforts to maintain body weight within 2% of baseline may unintentionally promote overhydration, particularly among slower athletes with lower sweat rates and greater fluid access [12].

Environmental stressors such as heat, humidity, altitude, and elevation changes further amplify hydration challenges. Studies in tropical and mountainous environments have shown that dehydration and hyponatremia can co-occur, particularly in athletes with salty sweat or those who rely on thirst alone in hot conditions [14,15]. Moreover, gastrointestinal disturbances, commonly reported in 30–50% of ultramarathon runners, may impair fluid and nutrient absorption, especially under thermal and psychological stress [18,19]. These symptoms, ranging from mild bloating and nausea to extreme vomiting, can disrupt hydration strategies even when fluids are available and planned appropriately.

Finally, logistic barriers such as aid station placement, course layout, and fluid type availability influence real-time hydration decisions. Even athletes with PFI strategies may be unable to follow them due to terrain difficulty, support crew limitations, or limited intake tolerance at high intensities [6,11].

Taken together, these factors illustrate that hydration during UER is not a simple matter of replacing what is lost. It is a dynamic, event-specific, individualized process requiring considerations of fluid access, environmental conditions, athletes’ physiology, and gut tolerance [20]. This complexity underpins the current debate over the efficacy of PFI versus TDFI and suggests that a hybrid or personalized approach may be most appropriate in this population.

## 3. Programmed Hydration Strategies

Programmed hydration refers to a prescriptive approach in which athletes aim to replace fluid losses based on estimated sweat rate, typically with the goal of preventing body mass loss greater than 2% [3,4]. These strategies are rooted in decades of laboratory-based research showing that exercise dehydration impairs aerobic performance, elevates core temperature, and increases cardiovascular strain, particularly during prolonged exercise in hot conditions lasting 60–120 min or more [3,4,16]. Such findings have led to formal guidelines recommending fluid intake sufficient to limit dehydration to under 2% body mass loss during endurance events.

The American College of Sports Medicine (ACSM) and the National Athletic Trainers’ Association (NATA) both recommend that athletes develop individualized hydration plans by calculating sweat rate during training and tailoring fluid intake accordingly to prevent excessive hypohydration and to maintain performance [3,4]. While this strategy is theoretically sound, ensuring replacement of sweat losses and preservation of plasma volume, it has been challenged in recent years due to its limitations in real-world endurance settings, particularly in UER [6,11].

One concern is that sweat rate is highly variable across individuals and environments and often misestimated, leading to mismatches between fluid needs and intake [16]. Additionally, body mass loss is not a perfect proxy for hydration status in prolonged ultra-endurance events, as it includes losses or gains from fuel oxidation, metabolic water production, and glycogen-associated water. For example, glycogen is stored with approximately 3–4 g of water per gram of carbohydrate, meaning that depletion of 400–600 g of glycogen could contribute to 1–2% body mass loss without any true fluid deficit [21]. Further mass loss occurs as the oxidized substrate is converted to carbon dioxide and water is exhaled, while metabolic water contributes to total fluid turnover without altering hydration status [22,23]. These processes explain how athletes can lose more than 2% of their body mass and yet remain clinically euhydrated, particularly when drinking according to physiological cues [10]. Programmed strategies based solely on body mass risk the promotion of overhydration, particularly in slower athletes with lower sweat rates who can consume more fluid than necessary due to longer aid station exposure [12].

Despite these concerns, laboratory studies do show performance benefits when hydration is maintained proactively. In a controlled trial, Jeker et al. [24] compared PFI with TDFI during 5 h of cycling in the heat. The PFI condition resulted in better power output and muscle force during a final time trial, as well as lower core temperature and greater plasma volume preservation. Similarly, Montain, Cheuvront, and Sawka [16] used modeling to demonstrate that fluid intake must be balanced with sweat sodium losses to avoid hyponatremia, even when total fluid losses are being replaced.

However, it is important to consider whether lab results accurately reflect what happens in real-world conditions. Wall et al. [25] conducted a blinded laboratory trial in which trained cyclists completed a 25 km time trial in the heat following IV induced 0%, −2%, or −3% hypohydration. It is important to note that this was an endurance, but not ultra-endurance, protocol. Performance was unaffected when participants were unaware of their hydration status, suggesting that psychological factors, rather than physiological impairment, may partly explain performance decrements reported in unblinded hypohydration studies. While this finding supports the idea that some hypohydration is tolerable, it also highlights the limitations of lab-based protocols in capturing real-world endurance dynamics.

Programmed strategies also assume consistent access to fluids and intake tolerance, which is often not the case in UER. Lavoue et al. [11] and Hoffman et al. [6] noted that despite individualized hydration plans, terrain, pacing, gastrointestinal tolerance, and aid station spacing frequently disrupt execution. The challenge is compounded in hot or technical races where gut function is compromised, and runners must balance fluid needs with carbohydrate and sodium intake [19].

Ultimately, PFI can be effective when based on accurate sweat testing and athlete experience, but its application in UER is limited by logistical, physiological, and perceptual concerns [20]. While it may provide structure and safety for some athletes, the evidence suggests that rigid adherence may be impractical and, in some cases, detrimental.

## 4. Drinking to Thirst

TDFI is a self-regulated hydration strategy in which athletes consume fluids based on their innate thirst response, rather than adhering to pre-determined intake volumes or weight-loss thresholds. This approach seems to have gained traction in ultra-endurance sports due to its simplicity, reduced risk of overhydration, and increased support from field-based ecological studies [9,10,12]. In contrast, PFI relies on estimated sweat rates and externally prescribed targets. TDFI assumes that thirst is an evolutionarily conserved and sufficiently reliable signal for fluid balance under most conditions.

Multiple field studies support the feasibility and safety of TDFI in ultra-endurance events. In a study of 383 participants in a 161 km trail ultramarathon, Hoffman and Stuempfle [12] found that 67% of athletes relied on thirst to guide hydration, and this group did not differ in finishing time or incidence of EAH compared to those following more structured plans. In fact, even among the fastest finishers, those in the top performance quartile, who experienced body mass losses exceeding 4%, no adverse outcomes were reported [12]. Similar observations were made in mountain and tropical events, where athletes who drank to thirst avoided gastrointestinal symptoms and completed races successfully, with moderate body mass loss and post-race hydration markers indicating no adverse fluid imbalance [14,15].

The physiological rationale for TDFI rests on the body’s ability to regulate plasma osmolality and blood volume through hormonal mechanisms [9]. As plasma osmolality rises, the thirst response is activated, promoting fluid intake that restores homeostasis without excessive consumption. This mechanism provides a responsive feedback system that adjusts to real-time internal demand, unlike programmed hydration, which may override these cues and lead to overdrinking [10].

Support for TDFI is further strengthened by evidence that moderate body mass loss does not impair performance, especially in self-paced, laboratory settings designed to mimic outdoor conditions. Wall et al. [25] conducted a blinded IV rehydration study in trained cyclists and found that up to 3% body mass loss had no effect on performance, core temperature, or perceived exertion. Goulet [26], in a meta-analysis, concluded that performance decrements from dehydration only appear in non-ecological laboratory protocols, and that in real-world endurance trials, TDFI may even enhance performance by reducing gastrointestinal burden and psychological strain. A common limitation in these studies, however, is that most are based on exercise protocols much shorter in duration than any UER.

A key limitation is the lack of structure in TDFI for athletes unfamiliar with their own fluid needs. Without a baseline understanding of sweat rate, sodium loss, or gastrointestinal tolerance, athletes may misjudge their intake, either underdrinking due to suppressed thirst or overdrinking when anxious about dehydration risk [18]. Thirst perception may be diminished under certain physiological or environmental conditions, such as cold exposure, hypoxia, or with aging, where plasma osmolality and hormonal signals lag behind fluid losses [3,7]. This delayed drive to drink can result in involuntary dehydration, even when fluids are available, highlighting the need for athlete awareness and proactive strategies to monitor hydration status. While TDFI works well for experienced athletes and in conditions where thirst cues remain intact, it may benefit from integration with pre-race education or training-based fluid testing to enhance accuracy and safety [5,8,20].

In summary, drinking to thirst appears to be a safe, practical, and physiologically sound strategy for many ultra-endurance runners, particularly those with experience, access to fluids, and self-awareness of their hydration needs. However, limitations, especially in less-experienced individuals or extreme conditions, highlight the potential value of hybrid approaches that combine internal cues with structured guidance.

## 5. Outcomes: Performance, Euhydration, and Safety

In UER, hydration strategies are primarily evaluated based on their ability to support performance, maintain euhydration, and prevent clinical complications such as hypohydration, EAH, and gastrointestinal distress. Both PFI and TDFI offer distinct advantages and limitations to achieving these outcomes. However, their effectiveness is highly dependent on individual physiology, environmental conditions, and race duration, making universal recommendations difficult. What works well for one athlete in a cool 50 km trail race may be ineffective or even harmful for another racing 100 miles through heat and elevation. This context-specific variability highlights the complexity of hydration in UER events and the need for flexible, individualized strategies. A summary of key studies can be found in Table 1.

### 5.1. Performance Outcomes

Hydration status plays a variable role in endurance performance, with outcomes heavily influenced by environmental stress, exercise duration, and whether trials are self-paced or fixed-intensity. In controlled lab settings, maintaining fluid balance through PFI can preserve plasma volume, reduce core temperature rise, and enhance late-stage performance. Jeker et al. [24] reported that cyclists on a programmed hydration plan performed significantly better in a 5 h heat protocol, with higher final power output and reduced muscle fatigue compared to a thirst-driven group. True conclusions drawn from these studies should be taken into consideration, but translation from lab or non-ultra-endurance events may not translate to UER events.

Conversely, evidence from internally valid laboratory studies and field-based ultra-endurance research suggests that performance appears to be largely unaffected by moderate dehydration [25]. Goulet [26] supported those findings with a meta-analysis, showing that self-paced endurance performance is typically not impaired until dehydration exceeds 3–4% and only in rare cases. Hoffman and Stuempfle [12] similarly observed no performance advantages for athletes who maintained a closer-to-baseline body mass versus those who lost more but finished in top positions.

The performance benefit of TDFI may lie in reduced gastrointestinal burden, fewer interruptions, and better alignment with internal perception. Athletes often report improved comfort, pacing, and focus when guided by thirst rather than external intake goals [6,20]. However, this depends on the athlete’s experience and their ability to accurately perceive and respond to hydration needs, which can be diminished in cold conditions or with gastrointestinal distress [17]. In addition to influencing comfort and performance, hydration strategies must also account for the prevention of serious clinical outcomes related to dehydration, EAH, and gastrointestinal dysfunction.

### 5.2. Euhydration and Body Mass Loss

Traditional definitions of euhydration during exercise are often based on maintaining body mass loss within 2% of pre-exercise weight, under the assumption that greater losses lead to hypohydration and compromised thermoregulation and cardiovascular function [3]. However, several field studies and expert reviews suggest euhydration may not align with body mass stability, especially in ultra-endurance contexts where fuel oxidation, glycogen-associated water loss, and metabolic substrate shifts can contribute to total mass loss [9,10,18]. In practice, runners often experience body mass losses of 2–5% or more, even with successful performance and no adverse symptoms [12,27].

Furthermore, weight loss alone does not reliably distinguish between dehydration and safe physiological adaptations, particularly when athletes drink to thirst and allow osmoregulatory mechanisms to guide intake [10]. In a tropical mountain ultramarathon, Belinchon-deMiguel et al. [13] observed that athletes experienced body mass losses of 3.7 ± 1.8% without signs of dehydration or EAH, suggesting that fluid losses were well tolerated and may be necessary for optimal performance. While body mass changes alone may not define hydration status, their influence on endurance performance remains an area of active investigation.

### 5.3. Health Risks and Clinical Complications

One of the strongest arguments in favor of individualized hydration strategies is the need to prevent clinical complications such as EAH, exertional heat illness, gastrointestinal symptoms, and cognitive or neuromuscular fatigue.

EAH is a well-documented risk in ultra-endurance events, especially when fluid intake exceeds sweat loss. Several studies report higher rates of EAH among athletes using programmed strategies or consuming sodium supplements with high fluid volumes [14,16]. Hoffman [10] emphasizes that sodium loss through sweat is rarely sufficient to warrant supplementation, and that normal race nutrition and drinking to thirst typically maintain adequate sodium levels.

Gastrointestinal distress also plays a pivotal role in hydration outcomes. Gastrointestinal symptoms are reported in up to 50% of ultramarathon runners, often due to the combination of high fluid, carbohydrate, and sodium intake [18,19]. Programmed hydration strategies that mandate intake at fixed intervals can exacerbate these symptoms, particularly under heat or fatigue, where gastric emptying and absorption are impaired.

TDFI strategies appear to reduce this burden by allowing athletes to self-regulate based on perceived gut tolerance, environmental cues, and availability, though they may not fully prevent underhydration if perception is blunted or inaccurate [17]. This underscores the need for athlete education and experience in developing a reliable hydration strategy that balances performance and health. For example, an athlete with a sweat rate of 2 L/h who underestimates their needs due to a suppressed or delayed thirst response may fall significantly behind on fluid intake in just a few hours. Once this deficit accumulates, the volume required to catch up may exceed gastrointestinal tolerance during running, leaving the athletes unable to rehydrate adequately without stopping, resting, and focusing entirely on fluid replacement, interruptions that clearly compromise performance. These scenarios highlight the importance of both understanding individual sweat rates and recognizing the limitations of thirst perception under prolonged stress. This supports the benefit of a hybrid hydration strategy of TDFI and PFI, in that there is a limit to overextending either strategy to a level that may compromise health and safety.

## 6. Synthesis and Future Directions

The current body of research highlights the complexity of hydration strategy selection in UER. While both PFI and TDFI have demonstrated utility, their effectiveness is highly context dependent. The optimal approach appears to lie not in strict adherence to either strategy, but in a more flexible, individualized model that integrates athlete experience, environmental conditions, and physiological variability.

The discrepancy between laboratory and field outcomes largely reflects the ecological constraints unique to UER. Laboratory protocols intentionally minimize variability to enhance scientific rigor, thereby limiting behavioral and environmental influences on hydration. In contrast, real-world events impose fluctuating intensities, unpredictable environmental conditions, and irregular aid station spacing that make strict replacement targets impractical.

Laboratory studies offer strong support for PFI, particularly in controlled, high heat conditions where sweat rates are elevated and fluid access is consistent. In these settings, typically lasting 2 to 5 h, PFI preserves plasma volume, reduces cardiovascular strain, and improves performance during submaximal endurance exercise [16,24]. However, these benefits often fail to translate directly to real-world ultra-endurance events, where unpredictable terrain, variable environmental stressors, gastrointestinal tolerance, and logistical limitations make rigid intake plans difficult to implement [6,11].

Conversely, TDFI aligns more closely with the realities of UER. Field studies consistently show that athletes drinking to thirst can perform at a high level, maintain adequate hydration status, and minimize the risk of overhydration and EAH [11,12]. However, TDFI is not without limitations. Some athletes underestimate fluid needs in cool conditions or when thirst cues are blunted, leading to underhydration or impaired recovery [17]. In rare cases, thirst may even fail to protect against overhydration. Armstrong et al. [28] documented two cases of EAH in cyclists who completed a 164 km ride in extreme heat and consumed over 13 L of fluid: both reported thirst of 1–2 on a 9-point scale at the finish line despite significant sodium dilution. This illustrates that thirst may not reliably signal fluid overload and underscores the need for experience and individualized guidance. Additionally, reliance on internal cues alone may be insufficient for novice athletes or those with little exposure to self-regulated fueling and hydration strategies [18].

This dichotomy underscores a central issue in hydration research; most recommendations are derived from protocols that lack ecological validity for UER scenarios. Studies employing fixed-intensity, short-duration, or non-blinded protocols may not reflect the physiological and behavioral realities of multi-hour, self-paced events [25,26]. There remains a clear need for studies conducted under race-like conditions, using ultra-endurance athletes as participants and real-time metrics to assess fluid balance, thermoregulation, performance, and perception.

Moving forward, the field should prioritize the development and testing of hybrid hydration models that combine the responsiveness of TDFI with the structure of PFI. Hybrid models aim to bridge the gap between laboratory precision and real-world adaptability, retaining PFI’s capacity for planned replacement while preserving the autonomy and situational flexibility characteristic of TDFI. These strategies might involve targeted athlete education, race-specific sweat testing, and real-time self-assessment tools to guide intake without enforcing rigid volume targets. This individualized approach is particularly important given the variability in sweat rate, sodium loss, gastrointestinal tolerance, and environmental exposure observed across ultra-endurance populations. In practice, hybrid strategies may combine pre-race sweat assessment with in-race responsiveness to thirst cues and environmental demands. Athletes can further refine their approach through post-race reflection, tracking body-mass changes, perceived exertion, and performance outcomes to dial in future hydration plans. Integrating wearable feedback and perceptual awareness could help athletes better align fluid intake with physiological needs across variable race conditions.

Future research should also explore technological innovations that allow athletes to monitor hydration status noninvasively during training and competition. Wearable sensors capable of tracking sweat rate or osmolality may provide feedback necessary to bridge the gap between prescriptive and perceptual hydration approaches. Additionally, studies investigating sex-based physiological differences, hormonal effects, and the impact of age and experience on hydration behavior would further refine best practices for diverse athlete populations. Furthermore, the influences of heat acclimatization, gastrointestinal tolerance, sex differences based on hormonal fluctuations, and macronutrient and electrolyte intake are beyond the scope of this review, but should be more thoroughly explored in UER. These all can influence hydration, and there is a paucity of research on interactions and UER for all of these factors.

Ultimately, current evidence suggests that a one-size-fits-all approach to hydration is neither practical nor safe in UER. Instead, a performance-focused, risk-aware, and athlete-centered strategy that integrates internal and external cues may offer the most effective path forward. Bridging the gap between field experience and laboratory science through ecologically valid research designs will be essential in developing the next generation of hydration guidelines, especially for UER.

## 7. Conclusions

Hydration strategy remains a critical but complex component of performance and safety in UER. This review highlights that while both PFI and TDFI have demonstrated value, neither approach alone is universally effective across all ultra-endurance contexts. Laboratory studies support PFI for preserving physiological function in hot, controlled conditions, yet field-based research consistently shows that TDFI is practical and effective under real-world race demands.

Ultra-endurance athletes face wide variability in sweat rate, sodium loss, gastrointestinal tolerance, environmental exposure, and pacing; all of which impact hydration needs and the ability to execute a fixed plan. Rigid adherence to prescribed fluid volumes can lead to overhydration and increase the risk of EAH, while reliance solely on thirst may not meet fluid needs. These findings underscore the importance of rejecting one-size-fits-all guidelines in favor of individualized strategies that respect both internal feedback and contextual demands.

Practical recommendations for athletes and coaches should center on education, experimental learning, and self-monitoring. Athletes may benefit from pre-race sweat rate assessments, simulated training in race-like conditions, and the development of intuitive hydration awareness. A hybrid approach, integrating programmed guidance with real-time responsiveness to thirst, environmental conditions, and gastrointestinal comfort, appears to offer the most adaptable and sustainable framework. As always, practicing scenarios that mimic race conditions offers valuable individual feedback.

As the field advances, research should prioritize ecologically valid studies in ultra-endurance settings, incorporating real-time physiological monitoring, athlete feedback, and diverse populations. Expanding hydration science beyond the laboratory and into the complexity of real-world ultra-endurance racing will be key to refining individualized hydration strategies that optimize performance and safety.

## Figures and Tables

**Table 1:** Summary of selected studies examining hydration strategies and outcomes on endurance and ultra-endurance performance.

References	Population/Event Type	Hydration Strategy	Outcome
Belinchon-deMigue et al. [13]	Mountain ultra runners from three independent events (n = 448; grouped < 45 km, 45–90 km, >90 km)	Observed/self-reported *ad libitum* (unprompted field questionnaire)	In the >90 km group, higher pre-race fluid intake and greater weekly training volume predicted faster performance (R^2^ = 0.23; *p* ≤ 0.01). No association between in-race fluid and finish time.
Arnaoutis et al. [14]	44 km Olympus marathon finishers (n = 62)	On-course *ad libitum*	EAH in 8% (5/62); hyponatremic runners consumed ~37% less Na^+^ (1.2 ± 0.3 vs. 1.9 ± 0.4 g) and ~21% less fluid (3.9 ± 0.6 vs. 4.9 ± 0.9 L); plasma osmolality rose only in eunatremic group.
Hoffman & Stuempfle [12]	161 km trail ultramarathon (n = 383 starters)	Observed; 67% drank to thirst; broad Na^+^ supplement use	≥2% BM loss is common, including in top finishers (mean ∆BM = −2.5 ± 1.4%); finish time not related to ∆BM (r = 0.06, *p* > 0.05); no EAH despite wide variation in Na^+^ use.
Lavoue et al. [11]	24 h World Championships (elite, n = 12, 11 finishers)	*ad libitum*; real-time logging	Fluid intake was 16.4 ± 4.6 L (0.68 ± 0.19 L·h^−1^); no EAH; performance positively correlated with energy intake (*ρ* = 0.674) and negatively with fluid volume (*ρ* = −0.776, *p* ≤ 0.05).
Garcia-Gimenez et al. [27]	Multi-stage ultra-trail (9 stages; 635 km; n = 4)	*ad libitum* (self-regulated; no prompting)	∆BM = −2.1 ± 0.5% in early stages; ad libitum strategy maintained hydration within safe limits; no EAH observed across stages.
Jeker et al. [24]	Laboratory cycling: 5 h fixed + 20 km TT (n = 8)	PFI (≤1–2% loss) vs. TDFI (randomized crossover)	Small physiological advantages with PFI (increased TT power ~5%, reduced core temperature ~0.3 °C, increased plasma volume ~3%. But overall time was similar when urination time included; no EAH.

**Abbreviations**: TDFI = thirst-driven fluid intake; PFI = programmed fluid intake; EID = exercise-induced dehydration; EAH = exercise-associated hyponatremia; TT = time trial; BM = body mass. **Note:** “Thirst-driven” indicates drinking only when thirsty. “Ad libitum” indicates freely available, unprompted fluid intake, and is used interchangeably where methodological prompting was minimal.

## Data Availability

No new data were created.
